# Pollen-Pistil Interaction in Response to Pollination Variants in Subtropical Japanese Plum (*Prunus salicina* Lindl.) Varieties

**DOI:** 10.3390/plants11223081

**Published:** 2022-11-14

**Authors:** Ankit Dongariyal, Dinesh Chandra Dimri, Pradeep Kumar, Ashok Choudhary, Priynka Kumari Jat, Boris Basile, Alessandro Mataffo, Giandomenico Corrado, Akath Singh

**Affiliations:** 1 Department of Horticulture, Govind Ballabh Pant University of Agriculture and Technology (GBPUAT), Pantnagar 263145, India; 2 Krishi Vigyan Kendra (GBPUAT, Pantnagar), via Guptkashi, Bansu, Rudraprayag 246439, India; 3 Division of Integrated Farming System, ICAR-Central Arid Zone Research Institute, Jodhpur 342003, India; 4 Department of Horticulture, Maharishi Arvind University, Mudiaramser, Jaipur 302012, India; 5 Department of Agricultural Sciences, University of Naples Federico II, 80055 Portici, Italy

**Keywords:** *Prunus salicina*, pollen germination, fertilization, pollination, fruit set, yield, self-incompatibility, sub-tropical

## Abstract

The Japanese plum (*Prunus salicina* Lindl.) is a fruit tree globally cultivated in temperate regions of the world. Its floral biology and yield are affected by several factors, with issues related to self- and cross- (in) compatibility among varieties being emblematic of the whole Rosaceae family. The aim of this work was to elucidate the fruit set, dynamics of pollen tube growth in pistil, and yield and other fruiting attributes, in ‘Satluj Purple’ and ‘Kala Amritsari’, probably the most popular subtropical Japanese plum varieties in northern regions of India. Specifically, we examined the response of six different pollination variants, namely to self-pollination, open-pollination with the two cultivars located in adjacent rows, open-pollination with the two cultivars located in distant rows, manual cross-pollination, supplementary pollination, and floral bouquet. During the two years of the investigation, both plum cultivars showed good in vitro pollen germination (on average, above 50%) at different sucrose concentrations, with the highest values for the ‘Satluj Purple’ and for the 15% concentration. In vivo, the analysis of the pollen growth in the various sections of the style indicated the best performance when pistils of ‘Satluj Purple’ were pollinated by pollen grains of cv. ‘Kala Amritsari’. Cross-pollination also registered faster growth of pollen tube in pistil with the lowest number of incompatible pollen tubes compared to open- and self-pollination. From the productive point of view, cross-pollination showed the most pronounced results among the different pollination variants, with the highest initial fruit set (36.6%) and yield (28.0 kg/tree), and the shorter fruit development in ‘Satluj Purple’ (fruit set and yield in self-pollinated ‘Satluj Purple’ trees were 3.3% and 2.0 kg/tree, respectively). Conversely, the use of ‘Satluj Purple’ pollen for ‘Kala Amritsari’ showed poor results. Finally, in our study, ‘Kala Amritsari’ showed self-compatibility. We conclude that the main cause of poor fruit set in ‘Satluj Purple’ is self-incompatibility. The relevant genotypic-specific effects revealed by the analysis of the various pollination treatments also highlighted the importance of interplanting to increase fruit set and yield for subtropical Japanese plum varieties.

## 1. Introduction

Plum is an important group of stone fruit trees and shrubs belonging to the genus *Prunus* (family Rosaceae). Plums are commercially cultivated for their fruits in numerous temperate regions of the world. With an average production of 229742 tonnes in 2020 and a 1.88% share in total world production, India ranks eighth among the world’s top plum-producing countries (FAOSTAT; www.fao.org; accessed on 21 October 2021). Besides its typical cultivation in hilly areas, some of the low chilling plum varieties (i.e., with chilling requirement below 300 h) are also grown in the subtropical climate of North-Western India [1]. In recent years, a significant reduction in chilling accumulation has been observed in temperate as well as subtropical climates [2]. The alarming indications of the Intergovernmental Panel on Climate Change of the United Nations (https://www.ipcc.ch/; accessed on 22 March 2022) point towards an increasing surface temperature, which is expected to alter plum phenological phases and species distribution [3]. Hence, the area under plum cultivation (e.g., for cultivars with lower chilling requirements) is changing worldwide and in India, it is expanding at a rapid pace, due to the hardy and precocious nature and adaptability of blooming patterns of plums [4].

Approximately 82% of the plum cultivated in Asia belongs to *P. salicina* (Lindl.), commonly known as Japanese or Chinese plum [5]. In India, the average productivity of plum (82.68 tonnes per hectare; FAOSTAT) is lower than that of the main plum-producing countries in the world. This is likely due to environmental as well as genetic factors, as observed in other fruit crops [6,7,8]. For Japanese plum in India, the underlying external factors for low productivity may include cultural practices, pest management, and incompatibility between different cultivated varieties. Like other stone fruit species, Japanese plum exhibits gametophytic self-incompatibility (GSI), a prezygotic reproductive barrier where genetically related pollen is rejected by the pistil [9,10,11]. In GSI, the growth of the pollen tube is arrested in the style region [8]. This incompatibility system is governed by a polymorphic locus (S) with two linked genes determining pollen and pistil phenotype [12,13]. Self-incompatibility occurs when the same S-allele is expressed in the haploid pollen grain and the diploid pistil [14].

Because of the economic impact of pollen–pistil interaction on fruit yield, this biological phenomenon has been studied in a range of plum cultivars in various counties [7,15,16,17,18,19]. ‘Satluj Purple’ and ‘Kala Amritsari’ are most likely the most widely cultivated plum varieties in Northern India. The cultivars are very popular, especially under subtropical conditions because of early fruit maturity, good fruit size, strong fruit coloration, and pleasant taste [20,21]. Nonetheless, especially for ‘Satluj Purple’, producers often complain about low fruit setting and yield [20,21]. These recurrent criticisms indirectly suggest that environmental and technical factors may not play a dominant role. ‘Satluj Purple’ is generally grown inter-planted with other cultivars (e.g., ‘Alu Bokhara’, ‘Kala Amritsari’, ‘Triton’) in commercial orchards but, to our knowledge, it is unknown whether issues related to pollen–pistil interaction are relevant. Pollen source is particularly important for self-incompatible genotypes [22] and understanding the pollination requirement of a cultivar is a crucial pre-requisite for orchard planning [11]. The most important pollen vectors for plum trees are honeybees (*Apis mellifera* L.) and bumblebees (*Bombus* spp.), while wind plays a negligible role [11].

Due to the economic importance of the ‘Satluj Purple’ cultivar in subtropical regions of India, the first objective of this study was to evaluate the self-incompatibility in this cultivar by elucidating the pattern of pollen–pistil interaction under self and cross-pollination with the ‘Kala Amritsari’ cultivar. Moreover, we aimed to establish the pollination requirements of ‘Satluj Purple’ and ‘Kala Amritsari’ under different pollination variants, and to examine their impact on fruit set, features, and yield. The outcome of the study can therefore provide useful information to increase yield in agriculture, revealing useful knowledge for orchard design, cultivar selection, and, ultimately, breeding.

## 2. Results

### 2.1. Differences in Flowering Period Are Limited and Are Appropriate for Cross-Pollination

**Figure 1:**
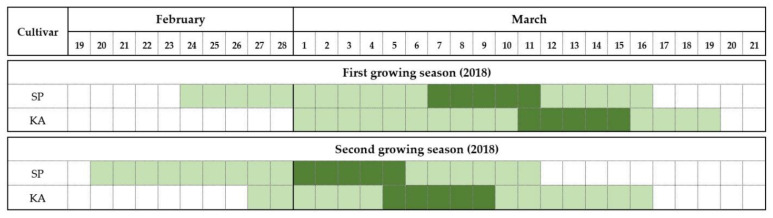
Flowering period of the two plum cultivars ‘Satluj Purple’ (SP) and ‘Kala Amritsari’ (KA) measured in 2018 and 2019. The light green indicates the flowering time, defined as the period between the stages “10% opened flowers” (BBCH stage-61) and “100% petal fall” (BBCH stage-69), whereas the dark green indicates the full bloom time defined the period when the percentage of opened flowers was between 50% (BBCH stage-65) and 70%.

The flowering phenology of ‘Satluj Purple’ and ‘Kala Amritsari’ trees is reported in Figure 1. In both years, the two cultivars had similar flowering duration that was approximately 18–20 days in both years. The onset of flowering in ‘Satluj Purple’ occurred on 24 and 20 February in 2018 and 2019, respectively. The flowering of ‘Kala Amritsari’ trees started on 1st March and 26 February in the first and second years, respectively. In both cultivars, the full bloom stage lasted five days. Although ‘Kala Amritsari’ flowered earlier than ‘Satluj Purple’, their flowering periods significantly overlapped.

### 2.2. In Vitro Assays of the Pollen Indicated a Good Germination Rate for Both Cultivars

**Figure 2:**
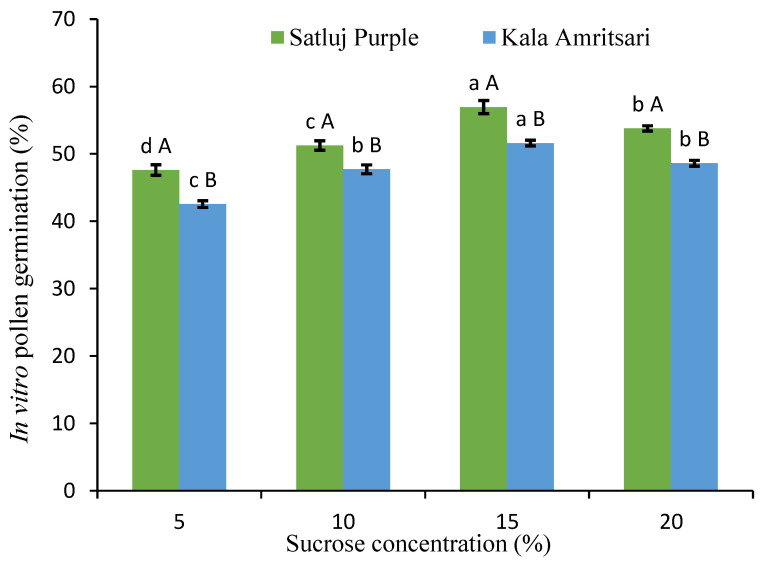
In vitro pollen germination under different sucrose concentration. Data are mean values from the 2018 and 2019 pollen. Separately for each cultivar, different lowercase letters indicate significant differences between sucrose concentrations according to the Tukey’s HSD test. Separately for each sucrose concentration, different uppercase letters indicate significant differences between sucrose concentrations according to the Tukey’s HSD test.

Having verified in vivo the overlap between the flowering period of the varieties, we aimed to assess the level and possible differences in pollen germination using an in vitro assay. The rate of pollen germination was always above 40% for both cultivars and every sucrose concentration (Figure 2). Significant differences in the in vitro pollen germination were present at all the sucrose concentrations, with pollen of the ‘Kala Amritsari’ having slightly lower germinability than that of the ‘Satluj Purple’. For both cultivars, the 15% sucrose concentration gave the highest pollen germination rate.

### 2.3. The Pollination Treatments Significanly Affect the Pollen Tube Growth Regardeless of the Variety

Having excluded large differences and cultivar specific deficiencies in pollen germinability, we focused on the possible presence of specific interactions in the various pollination treatments. The factorial analysis indicated that the pollination treatments and the year significantly impacted pollen tube growth in the style and ovary region, whereas these parameters were not affected by pollination treatment × year interaction (Table 1). The cross-pollination (P4) induced the highest number of pollen tubes in the upper style region, base of the style, and ovary region, while the lowest values of these parameters were found in self-pollinated trees (P1). The number of pollen tubes detected in upper third of the pistil style was higher in 2019 than in 2018, whereas the opposite results was found for the number of pollen tubes detected in the ovary (Table 1). Self-pollinated trees had the highest number of incompatible pollen tubes (i.e., the ones that failed to penetrate the ovary) followed by open-pollinated trees, whereas the lowest values of these parameters were measured in cross-pollinated trees. Most of the incompatible pollen tubes were observed in the lower half of the style region.

### 2.4. The Pollen-Pistil Interaction In Vivo Revealed Morphological Differences betwenn Self- and Cross-Pollination

**Figure 3:**
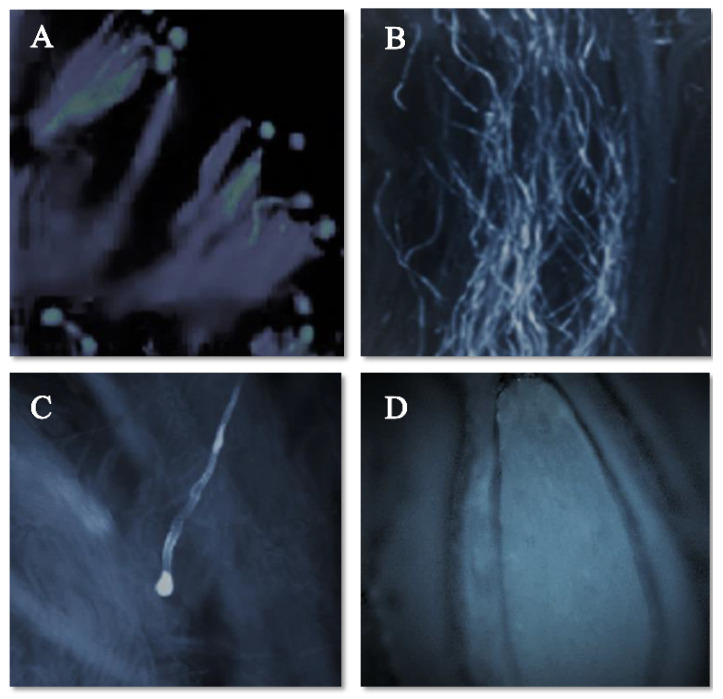
Pollen germination and pollen tube growth in pistil of the cultivar ‘Satluj Purple’ under Self-pollination (magnified 200×). (**A**) Pollen germination at stigmatic surface; (**B**) Pollen tube growth in the upper half of the style; (**C**) Inhibition of Pollen tube growth in the upper third portion of the style; (**D**) No entry of pollen tube in ovary region.

**Figure 4:**
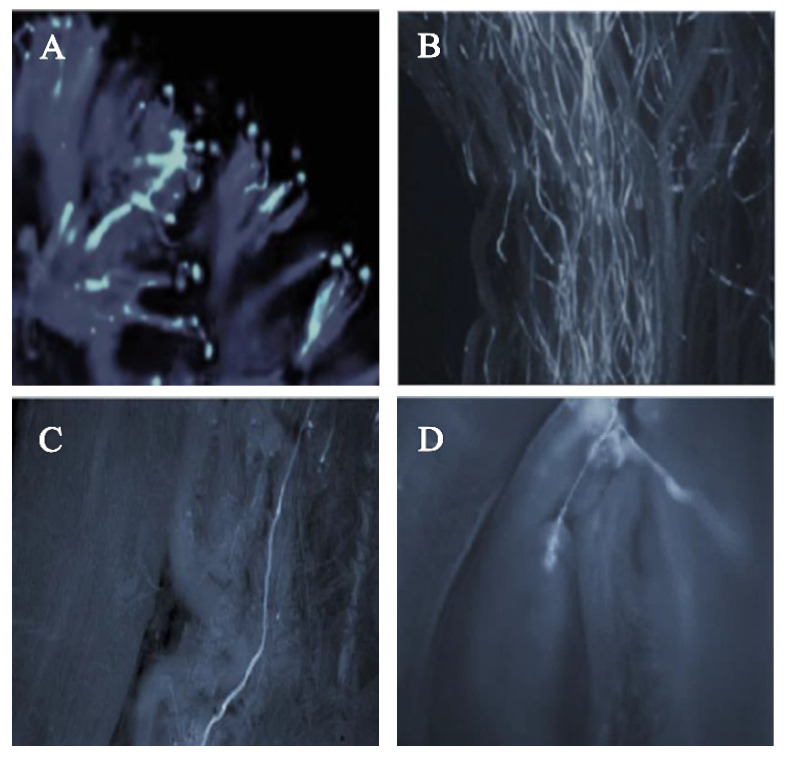
Pollen germination and pollen tube growth in pistil of the cultivar ‘Satluj Purple’ under cross-pollination with ‘Kala Amritsari’ (magnified 200×). (**A**) Pollen germination at stigmatic surface; (**B**) Pollen tube growth in the upper half of the style; (**C**) Pollen tube growth in the lower half of the style; (**D**) Pollen tube reaching the ovary region and penetrating one of the ovules of ‘Satluj Purple’.

The pollen–pistil interaction in the progamic phase starts with the germination of the pollen tube on the stigmatic surface as illustrated in Figure 3A (self-pollination) and Figure 4A (cross-pollination) for the ‘Satluj Purple’ variety. Pollen–pistil interaction begins with the germination of pollen grains at the stigmatic surface, as illustrated in Figure 3A (self-pollination) and Figure 4A (cross-pollination) for the ‘Satluj Purple’ variety. The florescent microscopy showed a gradual decrease in the number of pollen tubes from the top (stigmatic surface) to the base of the style. The number of pollen tubes with florescent callose was higher and clearly visible in the upper half of the style (Figure 3B and Figure 4B), which decreased in the middle half of the style in both self- and cross-pollination treatment (Figure 3C and Figure 4C). However, the morphological differences were observed in the growth of pollen tube for self- and cross-pollination regardless of the pollen donor. In self-pollination treatment, the pollen tube growth was arrested in the upper third portion of the style (Figure 3D). The pollen tube successfully reached to the ovary region in cross-pollinated trees (Figure 4D). Moreover, 240 h after the application of the pollination treatments, almost all the examined pistils had at least one pollen tube that reached the ovary, whereas in self-pollinated trees this percentage was around 50%.

### 2.5. The Pollination Treatment Affects Both Fruit Set and Plant Yield

#### 2.5.1. Initial Fruit Set, Final Fruit Retention and Yield

**Figure 5:**
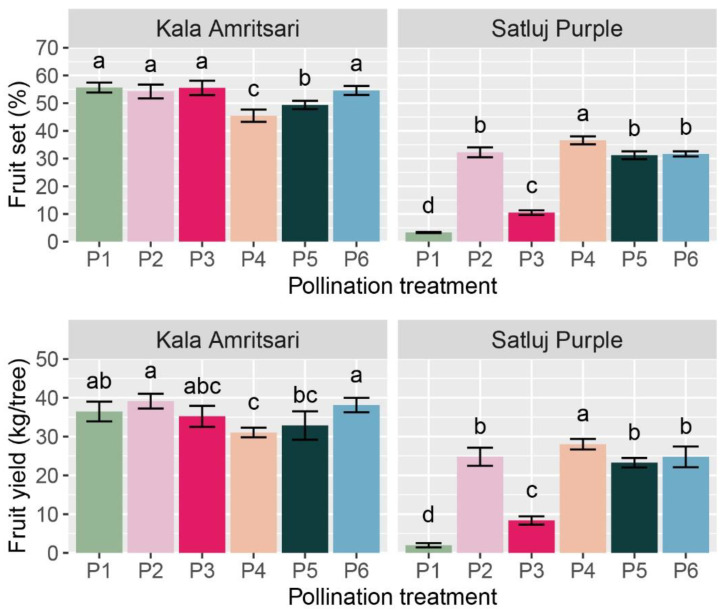
Fruit yield and fruit set of ‘Satluj Purple’ and ‘Kala Amritsari’ plum trees exposed to six pollination treatments: self-pollination (P1), open-pollination with the two cultivars located in adjacent rows (P2), open-pollination with the two cultivars located in distant rows (P3), manual cross-pollination (P4), supplementary pollination (P5) and floral bouquet (P6). For each parameter and separately for each cultivar, different letters indicate significant differences between pollination treatments according to the Tukey’s HSD test.

The cultivar and the pollination treatments had a significant impact on the initial fruit set (IFS), the final fruit retention (FF), and the fruit yield at harvest (Table 2). Independently of the year and the pollination treatment, ‘Kala Amritsari’ trees had fruit set, fruit retention, and fruit yield 116%, 44%, and 91% higher than ‘Satluj Purple’, respectively. Fruit retention was not affected by any of the interactions between main factors, and it was significantly lower in P1 (self-pollination) and P3 (open-pollination with the two cultivars located in distant rows) trees compared to the other treatments. Fruit set and fruit yield were significantly affected by the CV × P interaction (Table 2 and Figure 5). The effect of the pollination treatments on fruit set and yield was moderate in ‘Kala Amritsari’ trees, while it was highly in ‘Satluj Purple’ trees (Figure 5). In the latter, manual cross-pollination (P4) induced a 10- and 13-fold increases in fruit set and yield compared to self-pollinated trees (P1), respectively. A slightly lower increase in these parameters were induced by the P2, P5 and P6 pollination treatments (Figure 5). In the same cultivar, the P3 treatment induced a two- and three-fold increase in fruit set and yield, respectively. The self-pollinated trees of the two cultivars had large differences in fruit set (3.3% and 56% in ‘Satluj Purple’ and ‘Kala Amritsari’, respectively) and in fruit yield (2.0 and 36.5 kg/tree trees in ‘Satluj Purple’ and ‘Kala Amritsari’, respectively).

#### 2.5.2. Fruit Fresh Weight, and Growth and Development Duration

**Figure 6:**
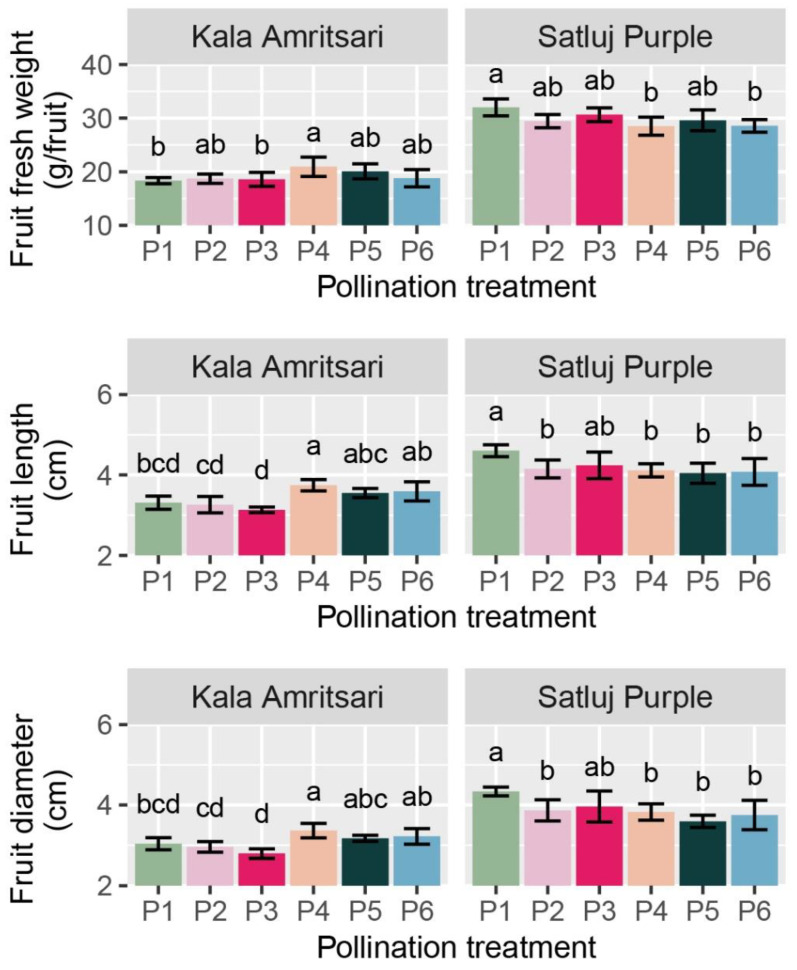
Fresh weight, length, and diameter of fruits of ‘Satluj Purple’ and ‘Kala Amritsari’ plum trees exposed to six pollination treatments: self-pollination (P1), open-pollination with the two cultivars located in adjacent rows (P2), open-pollination with the two cultivars located in distant rows (P3), manual cross-pollination (P4), supplementary pollination (P5) and floral bouquet (P6). For each parameter and separately for each cultivar, different letters indicate significant differences between pollination treatments according to the Tukey’s HSD test.

The fresh weight, length, and diameter of fruits were significantly affected by the cultivar and the C × P interaction, whereas these parameters did not differ between years (Table 2). Fruits of ‘Kala Amritsari’ were significantly smaller than those of ‘Satluj Purple’. In ‘Kala Amritsari’, P4 treatments induced an 8%, 13%, and 9% increases in fruit fresh weight, fruit length, and diameter compared to self-pollinated trees (P1), respectively, (Figure 6). Conversely, in ‘Kala Amritsari’, P4 and P6 induced significant decreases in these parameters compared to P1 trees (between approximately −11% and −13% depending on the parameter and the treatment) (Figure 6). Moreover, fruit length and diameter were also affected negatively by P2 and P5 compared to P1.

The fruit development period was on average five days shorter in ‘Satluj Purple’ than in ‘Kala Amritsari’ trees (Table 2). Independently of the cultivar and the year, trees exposed to the P2, P4, P5, and P6 pollination treatments had a slightly but significantly shorter fruit development (3–4 days) than in P1 and P3 trees.

### 2.6. Multidimensional Reduction of the Analysed Variables in the Pollination Variants Indicated a Predominant Role for the Genotype

**Figure 7:**
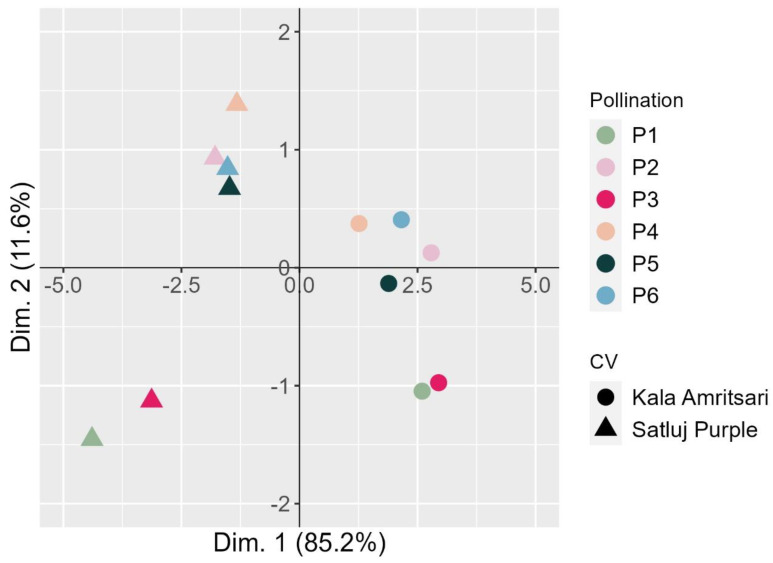
Bi-dimensional PCA plot of the fruit traits of two plum cultivars ‘Satluj Purple’ (triangles) and ‘Kala Amritsari’ (circles) exposed to six pollination treatments: self-pollination (P1), open-pollination with the two cultivars located in adjacent rows (P2), open-pollination with the two cultivars located in distant rows (P3), manual cross-pollination (P4), supplementary pollination (P5) and floral bouquet (P6). For both varieties, each pollination treatment is colored according to the bar reported on the right-hand side. The plot also reports the percentage of variance explained by each dimension.

A principal component analysis (PCA) was carried out to visualize the relationship of the various pollination treatments in the two cultivars (Figure 7). The first dimension clearly separated the two cultivars summarizing the main differences between the varieties in the measured traits (‘Kala Amritsari’ having higher fruit set, retention, fruit yield, but smaller fruits and shorter fruit development stage than ‘Satluj Purple’). The second dimension clearly separated the trees exposed to P1 and P3 treatments, describing mainly the shorter length of the fruit development stage induced by these two pollination treatments compared to the others.

## 3. Discussion

As other temperate fruit crops, each Japanese plum cultivar requires specific chilling and heat requirements during the fall–winter–spring period for breaking bud dormancy and, therefore, flowering/sprouting normally [11,23,24]. Similarly to other fruit trees species, the flowering time and duration in plum depend not only on the genotype, but are also influenced by the climatic conditions [11,25]. In our study, both cultivars advanced their flowering in the second season. The advancement in flowering in the second season may be attributed to the higher chilling accumulation in the fall and/or the warm temperature accumulated prior to the blooming period. Information on chilling and heat requirement of the main Japanese plum cultivars in India is limited. At least in the warmest countries around in the Mediterranean basin (e.g., Israel), early blooming is considered one of the reasons of low fertility [26]. Since, in our study, the two cultivars were grown in the same orchard (same environmental conditions), the fact that, in both years, ‘Satluj Purple’ bloomed consistently earlier than ‘Kala Amritsari’ suggests that the former has lower chilling and/or heat requirement than ‘Kala Amritsari’. Low-chilling cultivars often bloom earlier than high-chilling varieties when grown in the same environmental conditions [27]. Furthermore, a synchronized pattern of flowering period was also observed between the two cultivars along with overlapping at full bloom stage. Since a three-day overlapping flowering period is necessary to ensure effective pollination and fertility in stone fruit crops [28], the overlapped flowering period of the two studied plum cultivars can be considered sufficient for adequate pollination.

Pollen germination is an essential event for fertilization and directly reveals the pollen functionality of a genotype [29,30]. This phenomenon is highly dependent on the species, cultivar, and growing conditions and acts as an important contributor to successful fruit set in stone fruits [31]. It has been previously proposed that in plum a pollen germination of 25% can be considered a minimum threshold for ‘good’ pollen germination [32]. The results of our study showed that the studied cultivars exhibited pollen germination well above this threshold. Furthermore, in both cultivars, in vitro pollen germination showed a significant responsiveness to sucrose, which is in agreement with previous findings in plum [33]. Specifically, the pollen tube showed gradual elongation with the increase in the sucrose concentration in culture media up to 15% sucrose while pollen tube growth was retarded at 20% sucrose in culture media. These results are in accordance with the literature on various stone fruits [34,35,36].

Florescent microscopy is an efficient method for determining pollen–pistil interaction providing reliable information on the compatibility relationships in plum. Our results demonstrated that the highest number of pollen tubes in the style and ovary region were obtained in the cross-pollination, while self-pollination registered the lowest values followed by open-pollination variant, with a reduced number of pollen tubes in the lower half of the style. Despite the high number of pollen tubes in upper half of the style, the decreased number of pollen tubes in the lower half of the style in all pollination treatments could be due to the progressive narrowing of the transmitting tissue from stigma to ovary and digestion of pollen tube membrane by surrounding pistil tissues [7,15,37]. The pollen tubes reached the base of the style in 4–6 days depending on the pollination treatment. Previously, it has been reported in plum that the pollen tube requires 3–4 days for its growth along the style [15]. In our study, three days after pollination, the pollen tube growth was affected by the pollination treatment with maximum velocity of pollen tube in cross-pollinated flowers. Independently of the weather conditions, the velocity of pollen tube within the pistil is relatively lower in plum and apple compared to other pome and stone fruits [38]. A faster growth of pollen tube in cross-pollination treatment was also previously reported in plum [15,39], sour cherry [40], and sweet cherry [41]. In the self-pollination treatment, the growth of pollen tube was arrested in the upper third of the style with characteristic thickening and intense florescence due to the deposition of callose (β-1, 3-glucan) at the tip portion. This is consistent with previous studies showing the arrested growth of pollen tube in the upper half of the style in plum [7,16,19].

The yield of self-incompatible cultivars is highly dependent on presence of pollinizers in the orchard, and for this reason, the inter-planting of compatible pollinizers having synchronized flowering with main cultivar is indispensable in commercial orchards of self-incompatible cultivars of plum [28]. Self-pollination in ‘Satluj Purple’ resulted in low fruit set due self-incompatibility, whereas fruit set was improved by cross-pollination by ‘Kala Amritsari’. Moreover, fruit set varied with the distance of the ‘Kala Amritsari’ pollinizer. Supplementary pollination, floral bouquet placement, and open-pollination with adjacent row of pollinizer induced similar fruit settings. Previous studies reported that, in self-incompatible plum trees, fruit set sharply decreases when distance from the pollinizer increases from 10 to 20 m [42]. This has been associated with a preference of the bees for closer trees [42,43], under the hypothesis of energy maximization associated with the floral constancy behavior exhibited by pollinators [44]. In contrast, ‘Satluj Purple’ as a pollen source showed poor pollen–pistil interaction, resulting in the lowest fruit set in ‘Kala Amritsari’ trees. The significant initial fruit set from self-pollen in ‘Kala Amritsari’ indicates the high level of self-compatibility of this cultivar. The association between faster growth of pollen tube and self-pollination was also reported in self-compatible almond trees [45].

The literature regarding the comparative analysis of the effects of pollination strategy on fruit set and retention of plum cultivars is relatively scarce. In our study, results on final fruit retention elucidated that, in both cultivars, cross-pollination decreases fruitlets drop and this results in an increase in fruit yield. The intensity of fruit drop was more pronounced in ‘Satluj Purple’ compared to ‘Kala Amritsari’. Better fruit retention under cross-pollination probably derives from the fact that self-progeny often leads to less vigorous embryos, due to embryo degeneration and/or inbreeding depression [46,47,48]. However, ‘Satluj Purple’ and ‘Kala Amritsari’ exhibited different responses to self and cross pollen with respect to fruit yield, suggesting a prevalence of a genotypic specific inbreeding. In ‘Satluj Purple’, fruit yield was more related to the distance from the pollen source in both successive years of the study.

Physical attributes of fruit are economically important determinants of the market price and consumer preference. Besides the genetic makeup of a cultivar, fruit weight and size are affected by climate, crop load, bud quality, position of fruit in the flower and in the tree, vegetative and reproductive balance, and orchard management [49]. In both cultivars, our results on fruit weight and size are consistent with previous evidence that in Japanese plum, high crop load can affect negatively fruit weight and size at harvest because of intense fruit-to-fruit competition for resources [50,51]. The factorial analysis of the effect of the pollination treatment indicated that larger fruits were obtained in all the cultivar/pollination strategy combinations that induced higher crop loads (higher fruit set and/or fruit retention). Overall, a shorter fruit development period was observed in ‘Satluj Purple’ compared to ‘Kala Amritsari’. Furthermore, fruit development period was longer in all the pollination variants where self-pollen was used, while a trend of shorter fruit development period was observed in all outcrossed pollination variants in both the studied cultivars.

Finally, our study highlighted the importance and the magnitude of the effect of the incompatibility in relation to the yield of highly popular varieties. Therefore, further studies may be also extended to characterize the highly diverse germplasm under this perspective [17,25,52].

## 4. Materials and Methods

### 4.1. Plant Material

The experiment was carried out during two consecutive growing seasons, 2018 and 2019, at a plum orchard located at the Horticulture Research Centre (HRC), GB Pant University of Agriculture & Technology, Pantnagar (India) (29°50′ N, 79.3° E; 243 m above mean sea level). The experiment was performed on 15-year-old trees of two Japanese plum (*Prunus salicina* Lindl.) cultivars, ‘Satluj Purple’ and ‘Kala Amritsari’. Trees were grafted on plum seedling rootstocks, spaced 6 m × 6 m (corresponding to a planting density of around 278 trees/ha), and trained to an open vase system. ‘Satluj Purple’, also known as ‘Fla 1-2’, is a self-incompatible and early maturing selection from the University of Florida, introduced in India in 1979 [20]. ‘Kala Amritsari’ is an early maturing local selection [21]. These two cultivars were selected because of their significance and popularity in subtropical region of Northern India [20,21].

The climate at the experimental site is humid subtropical with dry summers and cold winters. Generally, the South-West monsoon commences in the second or third week of June and continues in appreciable strength until September, with its peak in July–August and few showers during winter and occasionally in summers. Mean daily temperature and weather data were provided by the agro-meteorological observatory located at Crop Research Centre (Pantnagar, India) are reported in the Figure S1 (2018) and Figure S2 (2019).

### 4.2. Experimental Design

The experiment compared two cultivars (‘Satluj Purple’ and ‘Kala Amritsari’) and six pollination treatments: self-pollination (P1), open-pollination with the two cultivars located in adjacent rows (P2), open-pollination with the two cultivars located in distant rows (P3), manual cross-pollination (P4), supplementary pollination (P5), and floral bouquet (P6). For each cultivar, four homogeneous trees were selected per treatment and used for the measurements. In the selected trees, 200 flowers were randomly selected at balloon stage (BBCH stage-61; see Section 4.3) and labelled. In the P1 treatment (self-pollination), the selected flowers in both the cultivars were bagged to prevent cross-pollination. In the P2 treatment, the two cultivars were in adjacent rows and selected trees were left for open pollination, whereas in the P3 treatment, trees of the two cultivars were located 6 rows (36 m) apart from each other. In the P4 treatment (manual cross-pollination), the selected flowers of both the cultivars were emasculated at balloon stage and were kept bagged until manual cross-pollination. In the P5 treatment (supplementary pollination), in addition to open-pollination, manual cross-pollination was conducted at anthesis (using the pollen of the other cultivar). In the P6 treatments (floral bouquet), branches of one of the cultivars were cut, dipped in water-filled bottles, and placed on the canopy of the other cultivar (four bottles per tree).

### 4.3. Flowering Phenology of the Two Cultivars

In both years, flowering phenology of the two cultivars was studied according to the BBCH Scale [53]. Ten trees per cultivar were daily and visually observed to estimate the percentage of opened flowers. Flowering time was defined as the period between BBCH stage-61 (10% opened flowers) and BBCH stage-69 (100% petal fall). Trees were considered at full bloom when the percentage of opened flowers was between 50% (BBCH stage-65) and 70%.

### 4.4. In Vitro Pollen Germination Assay

For in vitro pollen germination, 100 flowers were randomly collected at balloon stage from all around the canopy of three trees per cultivar. After removing sepals and petals, the anthers were isolated from the sampled flowers with the help of foreceps and needle and placed on the petri dish and stored under laboratory conditions at 20 °C until dehiscence (24–48 h). After dehiscence, pollen grains of both the cultivars were cultured in three petri dishes filled with germination medium containing 1% agar and different concentrations of sucrose (5%, 10%, 15%, and 20% *w*/*w*) and incubated (24 h at 20 ± 2 °C). After incubation, the pollen grains were observed under microscope (Olympus BX61, light field, magnified 10×). The number of germinated pollen grains was monitored under three different microscopic fields of view with each field encompassing 100 pollen grains per petri dish. Pollen grains having a pollen tube with a length larger than their diameter were classified as germinated [54].

### 4.5. Analysis of the Pollen-Pistil Interaction

The dynamics of pollen tube growth in the pistil of self-incompatible cultivar ‘Satluj Purple’ was examined in the trees of the P1, P2, and P4 treatments. Fifty pistils per treatment were collected and fixed in FPA solution (formalin:acetic acid:70% ethanol in a 1:1:18 ratio) and fixed for 72, 144, and 240 h. For histological study of pollen tube growth in pistil, the sample was gently washed in the running water followed by softening with 0.8 M NaOH for 6–12 h. After softening, the style region was separated from the ovary, making transverse and tangential sections of the ovule to make it easier to observe the pollen–pistil interaction in the micropyle and ovule region. The separated pistils were stained with aniline blue 1% (*v*/*v*) and examined under microscope (magnified 200×). Twenty pistils per fixation were analysed to examine quantitative parameters. The number of incompatible pollen tubes (i.e., the ones that failed to penetrate into the ovary) was counted and expressed as percentage based on cumulative value of pollen tubes in the upper part of the style. The number of pollen tubes in the upper third and base of the ovary and pollen tubes penetrating the ovary were counted separately. The region of pistil with pollen tube under different fixation periods (72, 144 and 240 h) was also analysed.

### 4.6. Fruit Set and Yield Components

In all the six pollination treatments, initial fruit set, fruit retention, and yield were measured on four one-year old shoots per selected tree (16 shoots per treatment). The number of fruits present after petal fall stage and those at harvest were counted and used to calculate the percent fruit set and fruit retention, respectively. Percent fruit set was computed by dividing the number of fruits at petal fall by the total number of selected flowers (×100), while fruit retention was calculated dividing the total number of fruits at harvest by initial number of fruit set (×100). Fruit fresh weight, fruit length, and diameter were measured on ten fruits per selected tree. In addition, the total number of fruits harvested per tree was counted. Fruit yield per tree was estimated multiplying the total number of fruits per tree by the mean fruit weight. The length of the fruit development period was calculated as the number of days between BBCH stage-65 (50% opened flowers) and harvest.

### 4.7. Statistical Analysis

The data collected in the experiment were subjected to ANOVA using the Tukey’s HSD (*p* < 0.05) as a post-hoc test for mean separation. These analyses were performed with SPSS statistical software package, version 28.0 (SPSS. Inc., Chicago, IL, USA). In addition, a principal component analysis (PCA) was carried out taking considering seven original variables (fruit set, fruit retention, fruit yield, fruit fresh weight, fruit length, fruit diameter, length of fruit development stage) using R 4.2.

## 5. Conclusions

Subtropical plum production in India is characterized by a less-than-optimal fruit set and yield. The lack of knowledge of adequate pollinizers (and related incompatibility) is often evoked as one of the most important and likely causes. This study provided experimental evidence of the impact the pollen–pistil interaction under self-, cross-, and open-pollination variants for the most important plum cultivar in India, ‘Satluj Purple’. Briefly, our work indicated genotypic-specific characteristics in their reproductive and production behavior. In summary, data indicated that cross-pollination has the most pronounced impact on pollen tube growth in style and ovary region with the lowest number of incompatible pollen tubes. Our work also indicated that, to increase fruit set and yield in the analyzed self-incompatible subtropical varieties, attention must be paid to the choice of pollinator. Specifically, cross-pollination along with supplementary pollination and floral bouquet placement conferred the best results for fruit set and yield in ‘Satluj Purple’. Future research will focus on the identification and characterization of compatible pollinizers with a higher synchronized flowering period in subtropical regions.

## Figures and Tables

**Table 1:** Significance of the effects of the pollination treatment (self-, cross-, and open-pollination), the year (2018–2019) and the pollination × year interaction (assessed with two-way ANOVA) on the number of pollen tubes—found in the sections of the upper third and the base of the pistil styles and of the ovary—and the number of incompatible pollen tubes in ‘Satluj Purple’ trees. Measurements were carried out 240 h after the application of the pollination treatments. Within each column and separately for the source of variation (pollination and year), mean values followed by the different letters represent significant differences (*p* < 0.05) according to Tukey’s HSD test.

Source of Variation	Number of Pollen Tubes			Number of Incompatible Pollen Tubes
	Upper Third of the Style	Base of the Style	Ovary	
Pollination (P)				
Self-pollination (P1)	48.37 ± 0.78 c	2.81 ± 0.32 c	0.37 ± 0.07 c	76.51 ± 0.92 a
Open-pollination (P2)	53.33 ± 1.09 b	11.16 ± 0.68 b	1.06 ± 0.03 b	16.59 ± 1.02 b
Cross-pollination (P4)	59.06 ± 1.24 a	21.09 ± 0.84 a	1.80 ± 0.11 a	6.48 ± 0.80 c
Significance	*	*	*	*
Year (Y)				
2018	51.61 ± 1.41 b	12.00 ± 2.63 a	0.97 ± 0.20 b	33.08 ± 10.85 a
2019	55.57 ± 1.78 a	11.38 ± 2.74 a	1.18 ± 0.22 a	33.31 ± 11.04 a
Significance	*	*	*	n.s.
P × Y	n.s.	n.s.	n.s.	n.s.

* and n.s. indicate significant differences at *p* ≤ 0.05 and not significant (*p* > 0.05) according to the two-way ANOVA, respectively.

**Table 2:** Significance of the effects of the cultivar, the year, the pollination treatment, and their interaction (assessed with three-way ANOVA) on the fruit set, retention, yield, fresh weight, length, and diameter, and on the length of the fruit development stage. Within each column and separately for the source of variation (cultivar, year, and pollination), mean values followed by the different letters represent significant differences (*p* < 0.05) according to Tukey’s HSD test.

Source of Variation	Fruit Set (%)	Fruit Retention (%)	Fruit Yield (kg/tree)	Fruit Fresh weight (g/fruit)	Fruit Length (cm)	Fruit Diameter (cm)	Length of Fruit Development (days)
Cultivar (CV)							
‘Kala Amritsari’	52.5 ± 0.7 a	44.6 ± 0.7 a	35.5 ± 0.6 a	19.2 ± 0.3 b	3.4 ± 0.1 b	3.1 ± 0.1 b	82.2 ± 0.3 a
‘Satluj Purple’	24.3 ± 2.1 b	31.0 ± 0.9 b	18.5 ± 1.7 b	29.8 ± 0.3 a	4.2 ± 0.1 a	3.9 ± 0.1 a	77.1 ± 0.4 b
Significance	***	***	***	***	***	***	***
Year (Y)							
2018	38.1 ± 2.9 a	37.5 ± 1.4 a	26.4 ± 1.8 b	24.7 ± 1.0 a	3.8 ± 0.1 a	3.4 ± 0.1 a	79.6 ± 0.6 a
2019	38.6 ± 2.9 a	38.1 ± 1.4 a	27.6 ± 2.0 a	24.3 ± 0.9 a	3.8 ± 0.1 a	3.5 ± 0.1 a	79.7 ± 0.5 a
Significance	n.s.	n.s.	*	n.s.	n.s.	n.s.	n.s.
Pollination (P)							
P1	29.5 ± 7.9 d	31.3 ± 2.6 b	19.2 ± 5.2 c	25.2 ± 2.1 a	4.0 ± 0.2 a	3.7 ± 0.2 a	82.3 ± 0.8 a
P2	43.2 ± 3.4 a	39.9 ± 2.2 a	32.0 ± 2.2 a	24.1 ± 1.6 a	3.7 ± 0.1 a	3.4 ± 0.1 ab	78.7 ± 0.9 b
P3	33.0 ± 6.8 c	33.5 ± 2.5 b	21.8 ± 4.1 c	24.6 ± 1.9 a	3.7 ± 0.2 a	3.4 ± 0.2 b	81.8 ± 0.7 a
P4	41.0 ± 1.4 b	42.2 ± 1.7 a	29.5 ± 0.6 ab	24.7 ± 1.2 a	3.9 ± 0.1 a	3.6 ± 0.1 ab	77.7 ± 0.8 b
P5	40.3 ± 2.8 b	39.6 ± 2.1 a	28.1 ± 1.6 b	24.8 ± 1.5 a	3.8 ± 0.1 a	3.4 ± 0.1 b	79.1 ± 0.9 b
P6	43.1 ± 3.5 a	40.2 ± 2.0 a	31.4 ± 2.1 a	23.7 ± 1.5 a	3.8 ± 0.1 a	3.5 ± 0.1 ab	78.4 ± 0.9 b
Significance	***	***	***	n.s.	n.s.	**	***
CV × Y	n.s.	n.s.	n.s.	n.s.	n.s.	n.s.	n.s.
CV × P	***	n.s.	***	***	***	***	n.s.
Y × P	n.s.	n.s.	n.s.	n.s.	n.s.	n.s.	n.s.
CV × Y × P	n.s.	n.s.	n.s.	n.s.	n.s.	n.s.	n.s.

*, **, ***, and n.s. indicate significant differences at *p* ≤ 0.05, *p* ≤ 0.01, *p* ≤ 0.001, and not significant (*p* > 0.05) according to the two-way ANOVA, respectively.

## Data Availability

The data not already included in the article that support the findings of this study are available on request from Ankit Dongariyal (A.D.).

## Supplementary Materials

The following supporting information can be downloaded at: https://www.mdpi.com/article/10.3390/plants11223081/s1, Figure S1: Daily mean temperature and weather data during 2018; Figure S2: Daily mean temperature and weather data during 2019.
