# Supplementary figures and images for: Pollen-Pistil Interaction in Response to Pollination Variants in Subtropical Japanese Plum (Prunus salicina Lindl.) Varieties
Source: Plants (Basel). 2022 Nov 14;11(22):3081. doi: 10.3390/plants11223081 (PMC9692414; doi:10.3390/plants11223081)

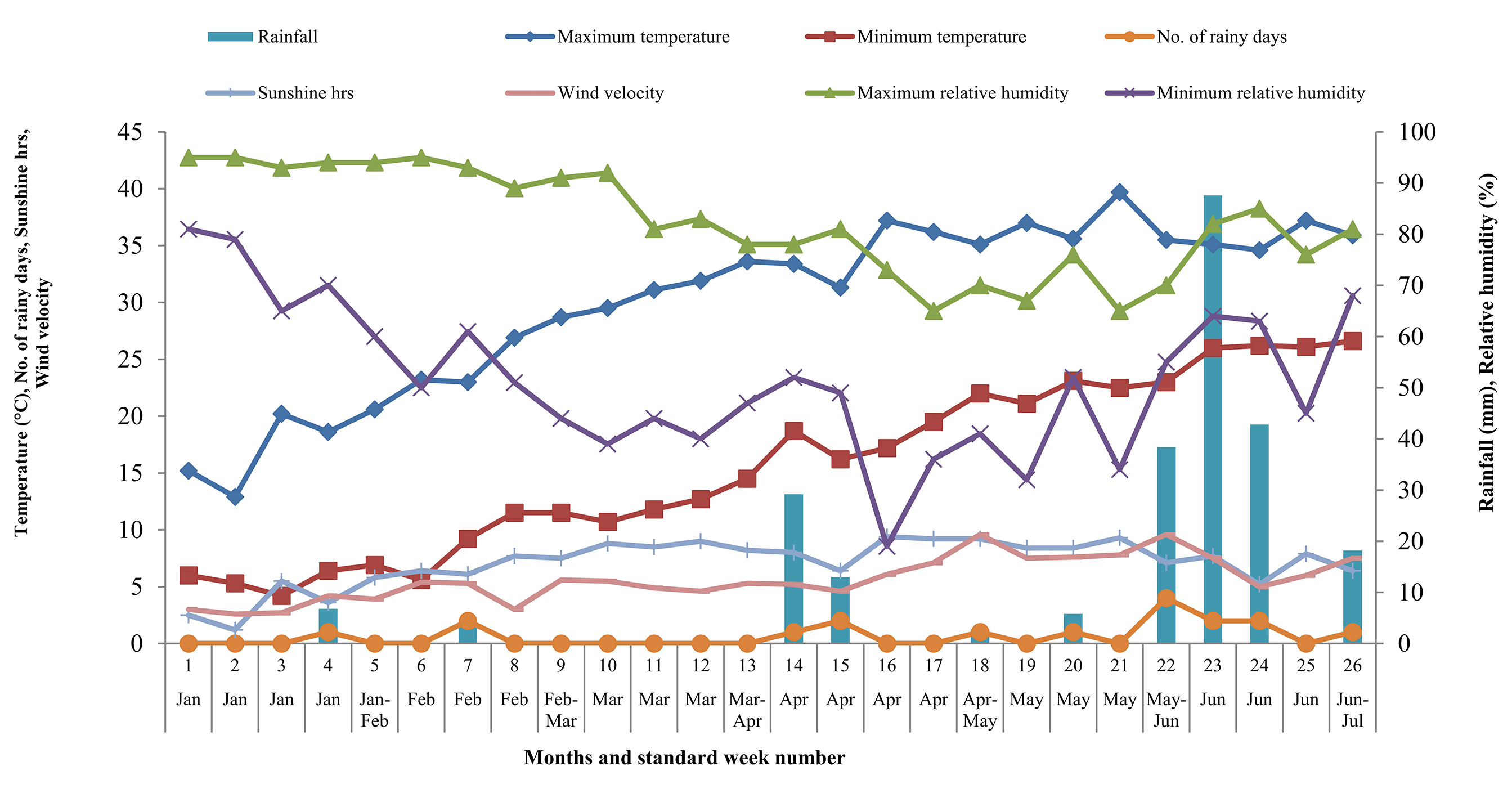

Supplement: Supplementary file 1 [file plants-11-03081-s001.zip › Figure S1.JPG]

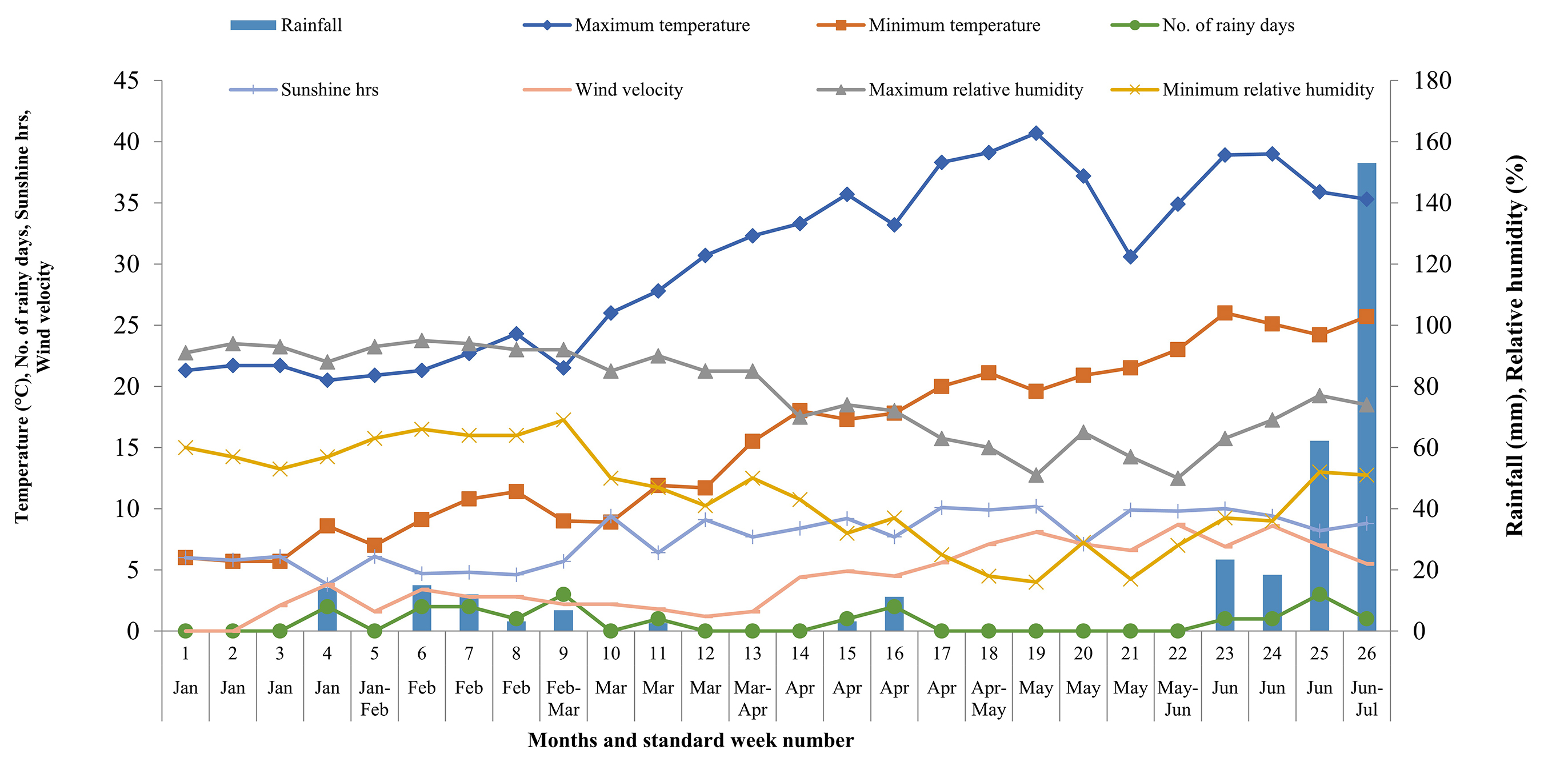

Supplement: Supplementary file 1 [file plants-11-03081-s001.zip › Figure S2.JPG]
